# A Thalamocortical Perspective on Sleep Spindle Alterations in Neurodevelopmental Disorders

**DOI:** 10.1007/s40675-024-00284-x

**Published:** 2024-03-11

**Authors:** Carolina Gutierrez Herrera, Leila Tarokh

**Affiliations:** 1 Department of Neurology, Inselspital, Bern University Hospital and University of Bern, Rosenbühlgasse 25, Bern, Switzerland; 2 Center for Experimental Neurology, Department of Neurology, Inselspital University Hospital Bern, University of Bern, Rosenbühlgasse 17, Bern, Switzerland; 3 Department of Biomedical Research (DBMR), Inselspital University Hospital Bern, University of Bern, Murtenstrasse 24 CH-3008 Bern, Bern, Switzerland; 4 Translational Research Center, University Hospital of Psychiatry and Psychotherapy, University of Bern, Bolligenstrasse 111, Haus A, 3000, Bern, Switzerland; 5 University Hospital of Child and Adolescent Psychiatry and Psychotherapy, University of Bern, Bolligenstrasse 111, Haus A, 3000, Bern, Switzerland

**Keywords:** Sleep spindles, Oscillatory activity, Neurodevelopmental disorders, Biomarker

## Abstract

**Purpose of Review:**

Neurodevelopmental disorders are a group of conditions that affect the development and function of the nervous system, typically arising early in life. These disorders can have various genetic, environmental, and/or neural underpinnings, which can impact the thalamocortical system. Sleep spindles, brief bursts of oscillatory activity that occur during NREM sleep, provide a unique in vivo measure of the thalamocortical system. In this manuscript, we review the development of the thalamocortical system and sleep spindles in rodent models and humans. We then utilize this as a foundation to discuss alterations in sleep spindle activity in four of the most pervasive neurodevelopmental disorders—intellectual disability, attention deficit hyperactivity disorder, autism, and schizophrenia.

**Recent Findings:**

Recent work in humans has shown alterations in sleep spindles across several neurodevelopmental disorders. Simultaneously, rodent models have elucidated the mechanisms which may underlie these deficits in spindle activity. This review merges recent findings from these two separate lines of research to draw conclusions about the pathogenesis of neurodevelopmental disorders.

**Summary:**

We speculate that deficits in the thalamocortical system associated with neurodevelopmental disorders are exquisitely reflected in sleep spindle activity. We propose that sleep spindles may represent a promising biomarker for drug discovery, risk stratification, and treatment monitoring.

## Introduction

Brain states are the manifestation of a delicate multilevel orchestration of activity and silencing of brain circuits required for each discrete state to function. While some circuits are state independent, others exhibit significant changes from one state to another. These changes are typically visualized using encephalographic recordings (EEG), which show the recruited cortical and subcortical networks’ constant activity. Wakefulness is characterized by low-amplitude, fast electrical signal oscillation (7–80 HZ), while non-rapid eye movement sleep (NREM) is characterized by high-amplitude, low-frequency slow oscillations (SO), and sleep spindles. NREM sleep can be ended by waking or entering rapid eye movement (REM) sleep, which is characterised by low-amplitude fast oscillations, transitory eye movements, and the absence of muscular tone. Humans, cats, and rodents have well-defined vigilance state transitions [1].

**Fig. 1:**
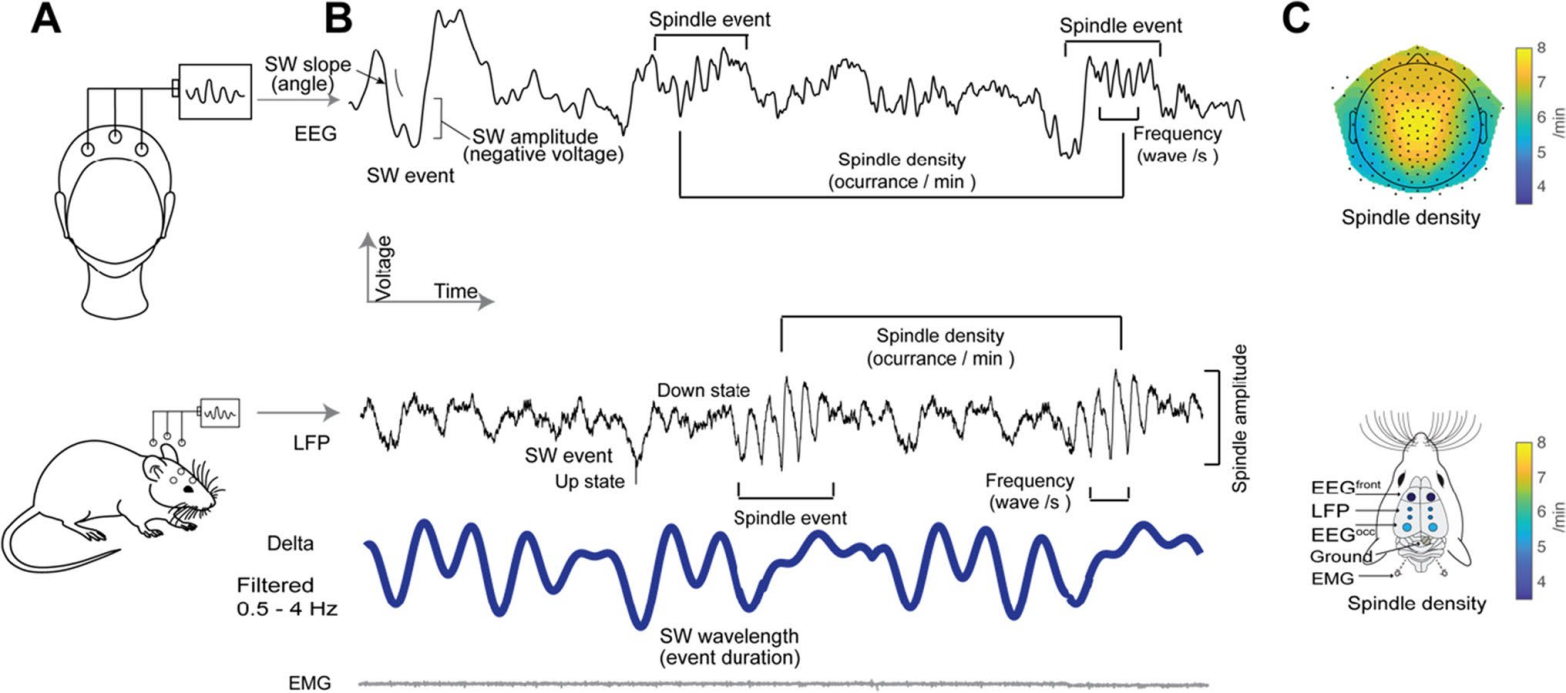
**A** Schematic of the typical human and mice EEG recording settings. **B** Representative human EEG (top) and local field potential signals (LFP) recorded from mice (bottom trace) displaying single slow wave events (SW), spindle events, the density (events per minute), and the typical number of cycles (frequency of the spindle event). The bottom blue trace is the delta power energy band (filtered between 0.5 and 4 Hz). Electromyogram (EMG), a measure of muscle tone, is shown below the blue trace. **C** Representative topographic plot from a high density (hd) EEG recording showing the typical topography of sleep spindle density (represented in spindles per minute) in a healthy adult human participant (top). Schematic of EEG and LFP electrode placement in mice showing representative quantification of the spindle density per electrode (bottom)

NREM sleep has fleeting waxing and waning oscillations in the 10–16 Hz range called sleep spindles (Fig. 1). Spindles were one of the first sleep activity patterns described in sleep research [2, 3]. Purpura’s recordings in sedated cats revealed that spindle events have an intra thalamic origin [4, 5]. This pioneering research set the stage for technological advances that enabled the recording of spindle activity in various animal species (for review, see [6]). However, the majority of sleep spindle research has focused on humans, cats, and rodents. Sleep Spindles have been proven to be functionally relevant in memory consolidation [7–12] maintaining sleep stability [11, 13–16] and to contribute to the proper development of cortical circuits from a physiological perspective [17–21]. In adult humans, spindles show topographic heterogeneity, which likely reflects the underlying thalamocortical connection. Mature fast spindles (13–15 Hz) are prominently detected in centroparietal locations often during the upstate of slow wave oscillations, whereas spindles detected in frontal cortices have lower frequencies (9–12 Hz) and occur on average 200 ms later than centrally detected spindles. These differences in spindle topography and dynamics are thought to be dependent on the underlying activity of the thalamocortical circuit and its developmental stages (see section below) [22–25]. We note that oscillations detected using EEG electrodes are highly conserved across species. However, the detection across animal models is more invasive, and the signals may differ when compared with superficial electrodes used in humans (see Fig. 1 for representative traces from humans and mice). Nevertheless, the definition of spindle activity between species remains generally conserved.

The generation and orchestration of spindle rhythms are associated with the recruitment of specific brain circuits [1]. While the mechanisms underlying the generation and dynamics of spindle oscillations have largely been elucidated, how this circuit develops and is modulated in humans and small animals is still the focus of numerous studies. Subtle oscillatory alterations are common in a variety of neurological and neuropsychiatric disorders suggesting shared underlying pathways or mechanisms. This review highlights key advances in circuit dissection and sleep spindle activity expression in humans and rodent models of neuropsychiatric disorders.

## Development of Sleep Spindles

### a. Evidence from Humans

The oscillatory activity of the brain can readily and non-invasively be measured using the EEG in even the youngest and most vulnerable populations. EEG electrodes about 1.5 cm in diameter are fixed on the scalp with a non-abrasive paste, and brain activity is measured from multiple brain sites. For sleep recordings, locations typically include central and occipital regions, though advances in EEG equipment allow for recording from 130 electrodes in preterm infants and up to 256 electrodes in toddlers. Among the oscillatory activity picked up by the EEG are delta brushes, which are a defining feature of the premature EEG [25–29]. These detectable electrical events with peaks typically falling between 8 and 20 Hz emerge in the EEG superimposed on delta waves (0.3 to 1.5 Hz) as early as 24 weeks to 34 weeks of gestation in humans based on data measured in preterm infants [25–27]. Their peak frequency, duration, and amplitude increase during development before decreasing with age (for review, see [26]). Low range synchronized activity and early gamma burst (EGB, 30–60 Hz) have also been reported [27], with spindle bursts (SB) temporally riding on the ascending phase of the delta brushes and gamma activity in infants [2–27]. Delta brushes are initially found only over central EEG derivations, but they subsequently spread to occipital and parietal regions after 28 weeks of gestation. Mature sleep spindles can be observed around four weeks after birth, but their occurrence during slow wave sleep (SWS) is infrequent, with approximately 3 or 4 spindles per hour [2, 3]. These spindles can be measured over central EEG derivations and are bilaterally synchronous. Over the following weeks, the expression of sleep spindles gradually increases, reaching an average density of about 4 events per minute during SWS by three months of age [30]. At two months, there is a noticeable peak in the sleep EEG spectrum within the spindle frequency range (11 to 16 Hz), which increases throughout the first year. Around four months of age, spindles can also be detected in frontotemporal regions, although they exhibit more asynchrony compared to central regions [22]. Spindles in their early stages (up to around six months of age) exhibit a distinctive trait of prolonged duration, lasting up to 15 to 20 s.

One distinguishing feature of immature spindles (up to approximately six months of age) is their long duration (as long as 15 to 20 s). During the first year of life, sleep spindles are diphasic—their morphology manifests a positive sharp component and a negative rounded component. Some authors have proposed that the shift in the sleep spindle’ morphology after the first year of life is driven by the well-documented shift in γ-aminobutyric acid (GABA) from an excitatory to an inhibitory neurotransmitter [31]. This intriguing hypothesis focuses on the role of neurotransmitter systems in the formation and maintenance of sleep spindles. Further details on the impact of neurotransmitter systems on spindle formation is provided in the subsequent section.

Spindle activity continues to increase throughout the pre-school years (ages 3 to 5 years) until the adolescent period [32]. Furthermore, sleep spindles become more coherent during early childhood within and across hemispheres, likely reflecting maturational increases in white matter connectivity during this age range [33]. Although data for middle childhood is sparse, existing evidence suggests that sleep spindle density increases during this time. Longitudinal data show that the amplitude of sleep spindles declines precipitously during adolescence, which is likely driven by the synaptic pruning that occurs during this developmental stage [34, 35]. Concurrent with this decline in amplitude, there is an increase in the frequency of sleep spindles, which has been suggested to reflect the increase in myelination during this developmental period [35]. Spindle density increases as well, reaching a trough around the age of 18. Spindles continue to become more coherent throughout adolescence [35]

In summary, spindle characteristics and occurrence vary across developmental stages from birth to adolescence, with spindle activity increasing, becoming more coherent and changing in amplitude and frequency as the brain continues to develop. These findings highlight the dynamic nature of spindle activity throughout development, shedding light on some of the critical underlying physiological processes and their potential treatment implications.

### b. Evidence from Animal Models

Sleep spindles are typically generated by the interaction of thalamic and cortical neurons [36]. Burst firing of the GABA cells of the thalamic reticular nucleus (TRN) [37] results in a rebound of activity of glutamatergic relay cells of the ventral posterior thalamic nucleus [38] (thalamocortical cells), which activate neocortical (or sensory cortical) layer five cortical cells. Cortical cells regulate the activity of the thalamic reticular nucleus (TRN) via the output corticothalamic layer 5/6 [39–42]. The cortical input to the thalamocortical networks is thought to modulate the termination of the burst of activity, which determines the spindle length [38, 43]. Further, the interplay between neurons and glial cells (e.g., astrocytes and microglia) has recently been suggested to play a role in sleep and spindle activity regulation [44–47]. Interestingly, alterations in glia populations have been linked to well-documented mental health disorders (for review, see [48–52]). However, the mechanisms by which they may be implicated are still not well investigated.

Other features, such as the intrinsic frequency (number of cycles/second) related to the burst discharge of TRN cells that defines a spindle and the refractory periods between (spindle density, number of spindles/minute) are regulated by intrinsic cellular properties (i.e., low threshold calcium (Ca2 +), voltage-gated calcium channels (Cav) 3.3 and Cav 3.2 channels and Ca2 + dependent potassium (K +) channels [53–57]). TRN bursts initiation is also modulated by intrinsic electrophysiological properties of the cells. In addition, synaptic-mediated activation of thalamocortical cells (TC) cells, rebound activity and electrical synchronization of the TRN activity via gap junctions, and the influence of various neurotransmitters like noradrenaline, acetylcholine, and serotonin will impact not only the expression but also spindle features like frequency, length, and amplitude. These mechanisms are elegantly review in [11].

Further research effort should be directed towards a better understanding which TC subserve different brain processes (e.g., memory, sensory processing, cognitive) and which underlying cellular and genetic mechanisms and/or microcircuits (neurons and glial cells) allow these functional differences considering neurodevelopmental disorders.

### c. The Role of Thalamocortical Circuit on Sleep Spindles Expression

The evolution of spindle maturation is dictated by the development of the thalamocortical circuits, which begins early in fetal life and continues throughout childhood and adolescence seamlessly [17, 59–62]. Cortical and thalamic circuits are thought to develop in parallel, and the emergence of mature brain oscillations such as slow waves, spindles, and gamma oscillations is dependent on the interactive and well-regulated communication between the thalamus and the cortex, as well as on the feedback from the cortex back to the thalamus [11, 61].

In brief, the development of thalamic circuits projecting to the cortex includes quantitative changes in thalamocortical neurons and concomitant increases in the strength of their connections with cortical cells [17, 55–58]. Thalamocortical projections into the cortex are determined, in turn, by their cortical inputs, which are driven by shared genetic programs, as is the case for sensory thalamocortical circuits formation and allow plasticity across sensory modalities [63–65]. In addition, the maturation of inhibitory interneurons in the cortex is critical in circuitry maturation [11, 59, 60, 65, 66]. A comprehensive review of the development of thalamic inhibition can be found in [11, 59].

Aside from the TRN, non-sensory thalamocortical networks including the intralaminar and central medial thalamus, anterior dorsal, and frontal cortices have received relatively less investigation. However, both animal models and human studies have demonstrated that sleep spindles are involved in the regulation of cognitive functions such as attention, executive control, and higher-order sensory processing [10, 14, 15, 66–69]. While there are signs of common regulatory processes with sensory networks, molecular, genetic investigations have found distinct components and signaling pathways that drive the formation of non-sensory thalamocortical circuits [11, 17, 60–62, 71, 72]. In humans, a study in adolescents examining the heritability of sleep spindles found a strong genetic contribution to posterior, but not anterior sleep spindles [58] supporting the notion that genetic factors contribute to spindle features. Still, the precise mechanisms involved in the evolution of sleep spindles, as well as their significance in sleep and brain development, remain unknown.

One of the challenges in translational approaches is the discrepancies in developmental timing and duration of brain maturation between animal models and the human brain. Regarding spindle maturation, this review primarily examines human and rodent data. To facilitate comparisons between humans and mice, we use the classification of Colonnese (2019) [59]. Based on this, thalamocortical development can be categorized into three broad stages: (1) the embryonic period, (2) the immature period, and (3) the mature activity period. These stages are reviewed in brief below.

### i. The Embryonic Period

The thalamus and cortex interact early in fetal development, forming the sleep spindle circuit. As the fetus grows, spontaneous motor activity may evoke associated neural activity, which guides neuronal differentiation, migration, synaptogenesis, and network formation [25–29]. In premature human neonates (29–31 weeks post conceptional age), delta brushes, triggered by spontaneous or induced motor activity to cortical activity, can be detected using a combination of scalp encephalography and monitoring of motor activity [27]. These are often localized to cortical sensory regions. Later, embryonic and perinatal spindle bursts are generated by motor activity bursts which can be detected in humans and rodents [19, 20, 70]. These may be unique to adult spindles since both events are identified in the sensory cortex, but separate TC circuits may be driving them. In addition to the somatosensory system, the medial thalamus-striatum and posterior geniculate nucleus (PGN) and Lateral geniculate nucleus (LGN) relay cells may be involved in delta brushes and spindle burst [6, 20, 71]. Clinical interest in neurodevelopmental pathology biomarkers has focused on neural correlates of these cortical events [72]*.*

### ii. The Immature Period

Experiments with cats and rats show that neurons in the cortical subplate play an instructive role in the generation of spindle bursts and thus guide proper cortical development. Given the fine tuning of the local circuits of the corticothalamic (CT)-TRN-TC, slight changes at the cellular and microcircuit levels could affect the overall dynamics of the TC loop, as shown in [15, 20]. The ventrobasal complex of the thalamus (first order of the sensory thalamus) and connectivity of this structure to the cortical subplate changes by migration of early developmental connections from cortical layers 5/6 to projections to layer 4 during postnatal (PN) 9–14 days in rats (corresponding to the childhood period in humans) [73]. Recent experiments in mice using intracellular recordings from the medial thalamus, sensory cortex, and striatum, showed that the expression of spindle burst density peaks around PN 9–12. Spindle bursts in the thalamus, on the other hand, are about half as frequent as earlier in development [18].

Evidence from animal experiments suggests that the topographical expression of cortical spindles may be regulated by the synchrony of different TC circuits [14, 74–77], and changes in their topography reflect the continued refinement/maturation of the thalamocortical circuits. Interestingly, thalamocortical interactions, measured by time lags of simultaneously recorded activity in the cortex and the thalamus (cross-correlations of activity) during development, are more prominent at lower frequencies (4–16 Hz) than correlations between the somatosensory cortex and other brain areas such as the striatum, which occur at 16–40 Hz [18]. Together, these findings suggest an inverse correlation between the maturation of spindles and cortical spindle bursts and the emergence of GABAergic inhibitory input in thalamic networks, albeit with different origins. Therefore, exploring the role of distinct brain regions which drive and coordinate burst activity and spindle generation at different developmental stages is key to understanding the temporal and spatial aspects of possible circuitry alterations that may give rise to the different neurodevelopmental disorders.

Spindle maturation is also associated with the development/differentiation of TRN. The TRN inhibitory projections to the thalamus act as a hub to control peripheral information traveling to the cortex by sending inhibitory projections to the ventrobasal thalamic nucleus (VB), to the dorsal thalamus, and to the anterior dorsal (AD)/anterioventral (AV), and anteromedial nucleus (AM) [14, 78–80]. However, it is still unclear if some of these projections are direct or collaterals. GABAergic parvalbumin (PV) positive cells are detected rostrocaudally and dorsoventrally of the nucleus at PN 0–4 [73, 81]. Further evidence in rats shows that GABA + cells are present in the TRN neuronal bodies, dendrites, and growth cones restricted to multi-vesicular bodies [61]. At this point, GABA released from growth cones depends on K + levels and is independent of Ca2 + [60]. Electron microscopy images from rat brains illustrate projections of TRN-PV cells to the medial part of the VB by PN 8. At PN 14, Nerve terminal distribution in the AD was more widespread than in the AV and anteromedial AM. By PN 21, axon bundles without nodular terminals in AV and AM were noted, showing a distribution of nerve terminals akin to the AD. Notably, the first connections from the TRN to AD form a link between the thalamus and the restrospinal cortex [61]. It is important to note that the nerve terminals at this stage have nodular properties, indicating a developmental uniqueness. [61].

### iii. The Mature Period

Slow spindles (11–13 Hz) at frontal, central, and parietal derivations during childhood mature to higher frequency fast spindles (13–15 Hz) at central parietal derivations during adolescence [17, 19, 32, 82]. Conversely, the density of sleep spindles (number of spindles per minute) peaks in late childhood before commencing a decline in adolescence. The decline in sleep spindles amplitude during adolescence is thought to be caused by synaptic pruning [35]. A similar evolution in slow wave characteristics occurs in rodents during the juvenile period (PN 21–35), which coincide with changes in synaptic density in the prefrontal cortex [83]. However, changes in the landscape of spindles expression have not been consistently reported.

By PN 21, changes in spindle expression correlate with topographic reorganization of the thalamic innervation into their corresponding mature cortical layers [61, 84]. Thalamic projections develop active synapses between PN 28 to PN35, and thalamic networks exhibit bi-stability allowing for the emergence of distinct EEG patterns during waking and sleep [70, 85–87]. In addition to these developmental changes in circuitry, major events in brain growth relevant to spindle expression occur. These include the increase in myelination across the brain concurrent with an initial increase in cortical synaptic density that peaks in late childhood (around 9 to 10 years of age) and then declines across the adolescent years [88].

The composition of GABA receptors is another determinant of changes in spindle expression [89–91], but detailed receptor structural composition across species is still limited. Furthermore, at different developmental stages, thalamic nuclei express distinct cell subpopulations. Expression of calbindin positive labeling (Calb) + and calretinin, for example, is detected in migrating cells in the medial thalamus and partially in the TRN, and it is correlated with the time course of thalamic neurogenesis. This is in contrast to the development of PV expression, which occurs first in the TRN and is correlated with the development of the CNS [89].

It is worth emphasizing that many anatomical studies on the development of the thalamocortical system have been performed in rats, cats, and more recently in mice (this is reviewed elsewhere [6]). While macroscopic EEG patterns are conserved across species [92], it is important to note that inter-species differences of the GABAergic populations (i.e., parvalbumin (PV)/somatostatin (SOM)/GABA/ glutamic acid decarboxylase (GAD)) have been reported in the TRN and thalamic networks (reviewed in [11]). Mice, for example, lack the interneural population in the TRN when compared to humans [92]. PV and GABA cells are densely labeled in the TRN and the AV of the guinea pig, but there is no PV innervation to the VB and the LGN. PV cells co-localize with Calb + in the TRN in the racoon, and GABA and GAD + cells seem to be restricted to the TRN similarly to human anatomical distributions [93]. Even though evidence strongly supports that thalamocortical maturation is essential for proper brain activity, subtle alterations in the inhibitory system and cortical connectivity may represent the neural basis of several neurodevelopmental and psychiatric disorders. Together, changes at the cellular, micro-circuitry, and circuit levels warrants the need for further studies on the temporal GABAergic system across different thalamic networks across different neurodevelopmental disorders.

## Functional Aspects of Spindles in Early Development

Among the functions of sleep spindles are the maintenance of sleep continuity and regulation of arousal (for review, see [11]). In addition, sleep spindles may play a crucial role in multiple neuronal network functions, such as memory consolidation and plasticity through dynamic alterations in synaptic plasticity [11], and may be a proxy measure for the individual’s learning potential [94, 95]. Sleep spindles have been associated with learning, memory, and intelligence in typically developing children [96]. However, little is known about spindles during infancy and the role they may serve in brain development. The morphology of early spindles differs from that of spindles found after the first year of life (reviewed above), suggesting that they serve diverse functions in brain and cognitive development. Recent longitudinal studies have correlated the transition from slow frontal expression in the early developmental ages to fast central-parietal expression in adolescents to improvements in memory consolidation [94]. Another study in infants found that sleep spindles occurring during quiet sleep are synchronized to limb twitches, and both show a developmental increase with age. The authors suggest that the development of functional connectivity among distant sensorimotor structures accounts for this synchrony, and spindles may play a role in establishing functional connectivity between cortical and subcortical regions [97]. However, the findings of this study are very preliminary due to the modest sample size and limited age range.

## Sleep Spindles in Neurodevelopmental Disorders

In this review, we address sleep spindles in four of the most prevalent neurodevelopmental disorders: intellectual disability, attention deficit hyperactivity disorder (ADHD), autism spectrum disorder (ASD), and schizophrenia (SCZ). Our aim is to provide a brief summary and synthesis of findings in humans and animal models regarding sleep spindles, as well as an outlook on the direction of future research [89].

### a. Intellectual Disability and Developmental Delay

Some functional aspects of the role of sleep spindle in cognition, memory, and stabilization of sleep have been inferred from developmental disorders characterized by changes in spindle expression. Although few studies have examined sleep spindles in children with intellectual disabilities, the existing literature supports alterations in spindle activity in these children. One limitation of interpreting these studies is that “intellectual disabilities” is an umbrella term encompassing a diverse array of disorders with varying neurobiological and genetic mechanisms. Here, we review some key studies.

In one study comparing children ages 2 to 6 years with developmental delay (*n* = 21) and typically developing children (*n* = 29), no difference in spindle density, frequency, or duration was found between the children with developmental delay and the typically developing (TD) group [98]. Another study comparing developmental dyslexia to TD children aged 7 to 16 years found that children with dyslexia had increased sigma power and increased spindle density [99]. Studies in children with Costello syndrome [100], a disease that results in malformations of cortical development, and 22q11.2 deletion syndrome [101], which is also associated with developmental delay, have also found increased spindling activity. On the other hand, most other studies have found reduced or even largely absent spindle activity in a wide variety of developmental disorders including cerebral palsy [102–104], late infantile neuronal ceroid [105] lipofuscinosis (LINCL), 15q11.2–13.1 [106], and Angelman syndrome [107]. Taken together, it is likely that different disorders (or even within a disorders class) impact the thalamocortical system in a variety of ways and give rise to a range of spindle features. What is clear is that in most individuals with intellectual disability, the thalamocortical system is impacted as reflected through sleep spindle activity. Whether these changes are etiological or reflect compensatory mechanisms needs to be investigated.

In efforts to investigate the relationship between developmental delay (DD), cognitive disabilities, and sleep spindles, animal models have highlighted relationships between DD and the expression of spindles. Remarkably, rats treated with a low-protein diet during early development—a model to induce DD—have fewer sleep spindles than litter mates that were kept under normal housing conditions. In a separate investigation, progeny from mother rats exposed to chronic sleep deprivation show signs of DD and a reduction in the number of sleep spindles [108], indicating that DD has a negative effect on sleep spindles and that sleep deprivation may exacerbate these negative effects. Disturbances in spindle density have also been observed in a mouse model of Angelman Syndrome (AS, Ube3a-del), a rare genetic disorder, that exhibits DD as well as impaired communication, motor and balance deficits, recurring seizures, and abnormal sleep patterns [109]. The loss of the neuronal-specific expression of UBE3A (ubiquitin-protein ligase E6-AP) is tallied with a decrease in the total NREM and REM sleep together with increases in the latency to REM sleep, similarly to those reported in AS patients. As a cross-section, these studies illustrate the links between brain development and the expression of cortically detected spindles, as well as the functional role of spindles in memory and cognitive development.

### b. Attention Deficit Hyperactivity Disorder (ADHD)

For the most part, studies in ADHD have found no difference in sleep spindle activity between individuals with ADHD and controls [110, 111]. These studies suggest that the thalamocortical system may not play a role in the manifestation of ADHD. ADHD serves as a compelling example of a neurodevelopmental disorder in which sleep spindles and, by extension, the thalamocortical system may remain unaltered. This highlights that sleep spindles represent a meaningful metric, showcasing alterations primarily within a subset of neurodevelopmental disorders.

### c. Autism Spectrum Disorder (ASD)

Few studies have thus far examined sleep neurophysiology in children with ASD, but consistent reports show that sleep spindles are markedly altered in ASD. A list of studies examining sleep spindles and their features in ASD can be found in Table 1; however, this is not a comprehensive list and other studies may exist. One small (*n* = 7 ASD; *n* = 13 typically developing) study of infants between aged 13 to 30 months found that the frequency of sleep spindles in children with ASD was lower than in typically developing (TD) children [112]. This finding was mirrored in a larger (*n* = 135 ASD; 29 TD) and older sample of 2 to 6-year-old children with and without ASD which found that children with ASD had diminished duration, density, and frequency of sleep spindles [113]. Similar findings have been reported in older children with ASD, with two studies reporting lower spindle density and diminished sigma power in ASD children when compared to typically developing children [98, 112, 114–119]. Furthermore, sleep spindle activity was associated with symptom severity in some of these studies. The overall picture is one of a consistent decline in sleep spindle density, with all but one study finding a reduction in this metric. As previously mentioned, spindle density is in large part related to the burst discharge of TRN cells and regulated by calcium and potassium-related channels. These findings, taken together, implicate the thalamocortical system in the etiology of ASD and are consistent with magnetic resonance imaging (MRI) studies in adults with ASD (e.g., [120]).

**Table 1:** An overview of studies which have examined sleep spindles in the neurodevelopmental disorders covered in this manuscript: Developmental Delay, Autism Spectrum Disorder, Attention Deficit Hyperactivity Disorder and Schizophrenia

	Amplitude	Frequency	Duration	Density	Sigma Power
**Developmental delay**					
Farmer et al., Neurology, 2018 (DD; n = 21; TD = 29), age range = 2–6 years	No change	No change	No change	No change	No change
Bruni et al., Sleep, 2009 (dyslexia,* n* = 16; TD = 11), age range (mean age: dyslexia = 10.8 and TD = 10.1 years)	NA	NA	NA	Increased	Increased
Smith et al., Developmental Science, 2017 (Dyslexia, *n* = 23; TD = 29), age range = 8–13 years (mean age: dyslexia = 10.09 and TD = 10.09 years)	NA	NA	NA	Trend increased	No Change
Della Marca et al., J Clin Neurophysiol, 2011 (Costello, *n* = 11; TD = 22), age range = 18 months–31 years (mean age: Costello = 9.6 and TD = 9.4 years)	NA	NA	NA	NA	Increased
Donnelly et al., eLife, 2022 (22q11.2DS, *n* = 28; TD Siblings, *n* = 17), age range = 6–20 years (mean age: 22q11.2DS = 14.6 and TD = 13.7 years)	Increased	Increased	No change	Increased	Increased
Shibagaki et al., Electroencephalogr Clin Neurophysiol, 1985 (cerebral palsy, *n* = 23; DD = 39), age range = 4 months to 5 years	NA	NA	Decreased	NA	NA
Shibagaki et al., Electroencephalogr Clin Neurophysiol, 1986 (cerebral palsy, *n* = 21; DD = 32), age range = 4 months to 12 years	NA	NA	NA	NA	Decreased
Veneselli et al., Brain and Development, 2001 (Neuronal ceroid lipofuscinoses, n = 18), age range = 1.6 to 6.6 years	Absent	Absent	Absent	Absent	Absent
Saravanapandian et al., Mol Autism., 2021 (15q11.2–13.1, *n* = 15, TD = 12), age range = 9 months to 13 years (mean age = 5.69 and TD = 5.78 years)	No change	NA	No change	Decreased	NA
Levin et al., Autism Res., 2022 (Angelman Syndrome, *n* = 12, DD = 12, TD = 12), age range = 10 months to 27 years (mean age across groups = 8.7 years)	No change	No change	Decreased	No change	Decreased
**Autism spectrum disorder (ASD)**					
Farmer et al., Neurology, 2018 (ASD; *n* = 85; TD = 29), age range = 2–6 years	No change	Decreased	Decreased	Decreased	No change
Godbout et al., Neuroreport, 2000 (ASD, *n* = 8; TD, *n* = 8), age range = 7–61 years	NA	NA	NA	Reduced	NA
Tessier et al., Int J Physiology, 2015 (ASD, *n* = 13, TD, *n* = 13), age range = 6–13 years (mean age: ASD = 10.13 and TD = 9.78 years)	NA	NA	Reduced	Reduced	Reduced
Mylonas et al., SLEEP, 2022 (ASD, *n* = 19; TD = 18), age range = 9–17 years (mean age: ASD = 13 and TD = 12)	No change	No change	No change	Reduced	NA
Page et al., Brain and Behavior, 2020 (ASD, *n* = 7; TD = 13), age range = 13 to 35 months; mean = 21.8 months	NA	NA	NA	NA	Reduced
Fletcher et al., Developmental Science, 2020 (ASD, *n* = 20; TD, *n* = 34), age range = 8 to 12 years (mean age: ASD = 125.5 months and TD = 118.9 months)	No change	NA	No change	No change	NA
Kurz et al., SLEEP, 2021 (ASD, *n* = 19; TD, *n* = 20), age range = 9 to 12 years)	NA	NA	NA	Reduced	NA
Limoges et al., Brain, 2005 (ASD, *n* = 16; TD, *n* = 16), mean age: ASD = 21.1 years, 21.8 years)	NA	NA	NA	NA	Reduced
Attention deficit hyperactivity disorder (ADHD)					
Prehn-Kristensen et al., PloS One, 2013 (ADHD, *n* = 16; TD, *n* = 16), age range = 9–12 years (mean age: ADHD = 10.6 and TD = 11.1 years)	NA	NA	NA	NA	No change
Prehn-Kristensen et al., Sleep Med, 2011 ADHD, *n* = 12; TD, *n* = 12), age range = 10–16 years (mean age: ADHD = 12.99 and TD = 12.64 years)	NA	NA	NA	No change	No change
**Schizophrenia**					
Buchmann et al., *Neuroimage,* 2014 (Scz, *n* = 21; HC, *n* = 21), mean age: Scz = 36 years, HC = 36 years)	No change	No change	No change	Reduced	No change
D'Agostino et al., *NPJ Schizophr.*, 2018 (Scz FDR, *n* = 16; HC = 16), mean age: Scz FDR = 48.5 years, HC = 49.8 years)	No change	No change	No change	No change	No change (reduced integrated sleep activity)
Ferrarelli et al., *Am J Psychiatry,* 2010 (Scz, *n* = 49; HC = 29), mean age: Scz = 38.2 years, HC = 36.7 years); range = 18 to 55 years	Reduced	NA	Reduced	Reduced	Reduced
Ferrarelli et al., *Am J Psychiatry,* 2007 (Scz, *n* = 18; HC = 17), mean age: Scz = 38.2 years, HC = 36.7 years); range = 18 to 55 years	Reduced	NA	Reduced	Reduced	Reduced
Kaskie et al., *Journal of Psychiatric Research,* 2019 (FEP Scz,* n* = 27; HC = 23), mean age: FEP Scz = 23.2 years, HC = 24.7 years)	No change	NA	Reduced	Reduced	NA
Manoach et al., *Front Human Neuro,* 2014 (Scz, *n* = 26; HC = 25), mean age: Scz = 27 years, HC = 27 years)	No change	Reduced	No change	Reduced	Reduced
Sasidhran et al., *Sleep Medicine*, 2017 (Scz, *n* = 45; HC = 39), mean age: Scz = 27.8 years, HC = 27.6 years)	NA	NA	NA	Reduced	Reduced
Schilling et al., *Eur Arch Psych Clin Neuro*, 2016 (Scz, *n* = 17; HC = 17), mean age: Scz = 29.9 years, HC = 26.5 years)	Reduced	No change	Trend	Reduced	NA
Wamsley et al., *Biological Psychiatry*, 2013 (Scz, *n* = 21; HC = 17), mean age: Scz = 34 years, HC = 36 years)	No change	No change	No change	Reduced	Reduced
Markovic et al., *Schz Research*, 2020 (Scz, *n* = 17; HC = 17), mean age: Scz = 16 years, HC = 16 years) Age range = 9–21 years	Reduced	Reduced	Reduced	Reduced	Reduced

While animal studies investigating sleep and spindle expression in models of autism are limited, a representative study in a rat model of autism linked brain connectivity during early development with reduced sleep spindles [121]. This mouse model shows behaviors which mirror those of ASD children, including deficits in social interaction and novelty seeking. This study suggests that alterations in circuit connectivity during development, specifically in excitatory/inhibitory balance, may account for the deficits in spindle expression and sensory processing found in ASD. Supporting this hypothesis, R-baclofen, a GABAB agonist, recovered the sensory processing deficit involving neuronal excitation/inhibition in the Cntnap2 Knock-Out rat model in a dose-dependent manner [122].

Moreover, in a mouse model of fragile X syndrome (FXS; dfmr1 and Fmr1 knockout (KO)/Fxr2 heterozygote) where circadian rhythmicity is affected, interictal epileptiform discharges (IEDs) appear in the EEG, indicating a common dysregulation of the balance between inhibitory and excitatory tone in cortical computations [123, 124]. These claims are supported further by the Gabra5 − / − mouse model, which lacks a-subunit 5 of GABA A receptors, where spindle expression is diminished to wildtype control litter mates, 0–2 Hz EEG activity is enhanced across states, and 8–12 Hz is decreased during REM. All of this lends credence to the hypothesis that alterations in excitation determined by alteration of the ratio between excitatory/inhibitory neuronal activity (E/I) due to the loss of GABA subtype A receptors may contribute to sleep and spindle deficits in ASD models [125].

Interestingly, it is posited in Asperger’s syndromes changes in sleep spindle intrinsic frequency in different brain regions may result from immaturity in brain regions related to the integration of sleep spindles, and this immaturity could be related to cognitive aspects in these patients. In support of this claim, advanced computational approaches were used to classify large genetic data bases of Asperger’s, researchers discovered that genetic regulation, synaptic transmission affecting excitatory/inhibitory tone, cellular calcium signaling, and short/long range circuit connectivity and networks all occur during the early postnatal period. One of the neuronal populations most affected by schizophrenia is that which expresses the Ca2 + -binding protein parvalbumin (PV). PV expression is often reduced at both messenger RNA (mRNA) and protein levels in human ASD brain samples and mouse ASD (and schizophrenia [15, 126, 127]) models. This leads to the hypothesis that neurons at the PV level may be causally related to the etiology of ASD and could also contribute to the alterations in inhibitory tone in thalamocortical networks in other psychiatric disorders such as schizophrenia.

### d. Schizophrenia

Sleep spindles deficiencies have been consistently observed in adults with schizophrenia (reviewed in [128–130]) as well as in children and adolescents with childhood onset schizophrenia (see Table 1). A recent meta-analysis reported a decline in sleep spindle density across studies with a large effect size, which was correlated with illness duration [131]. Another recent systematic review and meta-analysis reported that sleep spindle amplitude, density, and duration were reduced in both early and chronic psychosis [132]. Diminished sleep spindles have also been reported in first-degree relatives of patients with schizophrenia. Polygenetic risk scores for schizophrenia were associated with higher fast spindle amplitude, density, and intensity in a community sample of adolescents [133]. A correlation between sleep spindle density and left mediodorsal thalamic volume was found in a study of adults with schizophrenia, which measured both sleep EEG and structural MRI volume [134]. This study found that individuals with schizophrenia had lower sleep spindle density and medial thalamic volume compared to healthy controls. These findings suggest that thalamic volume may underlie some of the deficits in sleep spindles seen in schizophrenia. Table 1 summarizes a selection of studies examining sleep spindles in patients with schizophrenia; however, because there have been several recent reviews and meta-analyses on this topic (see [8, 135–139]) this is not an exhaustive list. The overall findings from these studies show that sleep spindle density is consistently diminished in individuals with schizophrenia as compared to those without schizophrenia. This is similar to the picture emerging in ASD, where sleep spindle density is the most consistent finding reported across studies. This overlap between ASD and schizophrenia is unsurprising given that both disorders have thalamocortical deficits and have a genetic overlap [135]. Collectively, these findings indicate that alterations in sleep spindles may be a risk marker for schizophrenia and may have a genetic basis.

Recently, rodent models have fueled prominent hypotheses on the pathogenesis of schizophrenia including deficits in dopamine, glutamate/N-methyl-D-aspartate (NMDA) regulation, alterations in neuroimmune/neuroinflammatory responses, deficits in GABA tone, and more recently dysregulation of the oxidative stress system (reviewed in [140–142]). In rodent models, the deficits in oscillatory activity including spindles could represent strong biomarkers linking circuit specificity and system alterations fueling discovery. This section aims to highlight recent findings on the possible circuit alterations that may underpin the sleep spindle deficits in rodent models of schizophrenia.

Experiments in rats with the NMDA glutamate receptor antagonist ketamine show a decrease in sleep spindle and delta power in fronto-parietal EEG, as well as an increase in higher frequency oscillations such as gamma power (30–80 Hz) [143]. A mouse model with Gria1 gene knock out (KO), which codes for the GluA1 subunit of α-amino-3-hydroxy-5-methyl-4-isoxazolepropionic acid (AMPA) receptor, supported these findings, reporting a decrease in sleep spindle activity in Gria1KO mice [136]. Concurrent to the decline in sleep spindle activity, an increase in slow wave activity (0.5–4 Hz) restricted to the occipital region was found. A mouse model with dysregulation in the oxidative stress system provides additional evidence. These mice have a deletion in one of the subunits of the glutathione synthetase (Gclm KO), which causes developmental reduction on PV positive cells in the TRN and anterior cingulate cortex early in development, followed by other brain areas (i.e., hippocampus and amygdala [144]). Electrophysiological recordings of KO mice showed an increase in delta power across of sensory and non-sensory TC networks, including the TRN, anterior and sensory cortex. 

Surprisingly, despite prolonged wakefulness leading to heightened oxidative stress due to mild sleep deprivation [145], knockout mice exhibit no alterations in sleep onset latency, increased delta power in subsequent non-rapid eye movement (NREM) sleep episodes, or heightened spindle density—features typically associated with sleep homeostatic responses. Treatment with a clinically relevant antioxidant rescue sleep alterations and spindle deficits suggests that oxidative stress affects susceptible networks that may be involved in sleep disturbance including sleep fragmentation and the regulation of spindle activity through TC networks.

More recently computational models, which simulate the dynamics of the thalamocortical system based on the anatomy and functionality of this network, have complimented work in experimental models to yield novel insights into the modulation of various spindle features by thalamocortical circuits. Lavarone et al. [146] exemplify the effectiveness of this approach by demonstrating that the interactions within thalamo-reticular networks change during different states of vigilance. Furthermore, the study emphasizes the crucial role of thalamic network interactions in the waxing and waning of spindles and suggests that alterations in thalamic excitability may influence the frequency and occurrence of spindle oscillations, which are relevant to certain mental disorders.

## Conclusion and Future Perspectives

**Fig. 2:**
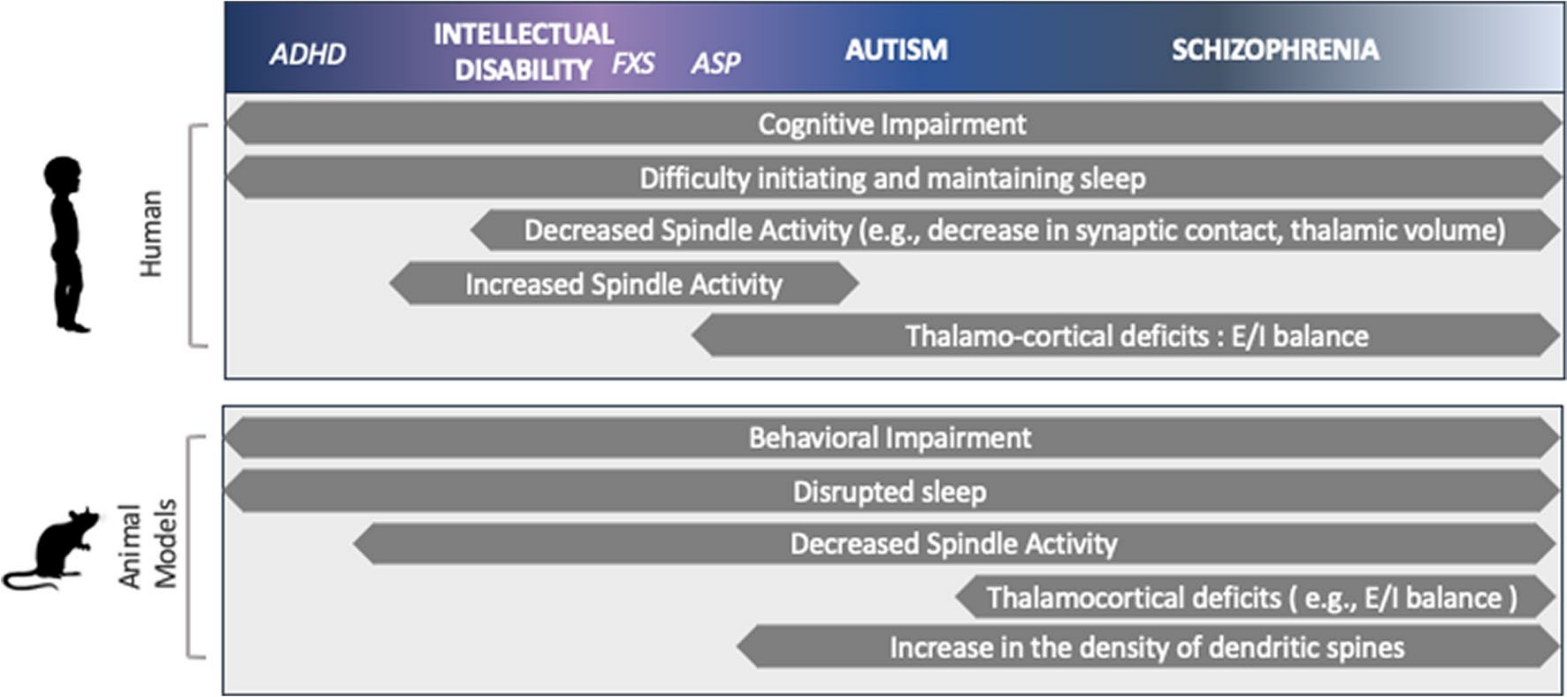
Summary of the common traits shared between humans and animal models of neurodevelopmental disorders. Common to both humans and animal models of neurodevelopmental disorders are cognitive/behavioral impairment, disrupted sleep, and alterations in spindle activity. Neurodevelopmental disorders are listed at the top and shown as existing on a continuum, with overlaps in symptoms and neurobiological underpinnings

Neurodevelopmental and neuropsychiatric disorders manifest as a wide range of structural, functional, and clinical abnormalities. Common among neurodevelopmental disorders are alterations in the thalamocortical network, as this network is responsible for cohesively relaying sensory information from the periphery to the cortex. Sleep spindles expression provides an elegant in vivo correlative measure of thalamocortical network integrity. Additionally, spindle features, such as density, frequency, and length, have been linked to the activity of various thalamocortical circuit dynamics. These are summarized in Fig. 2. An effective approach to understand the neural foundation of these neurodevelopmental disorders involves a meticulous analysis of the characteristics of sleep spindles. Despite the mounting evidence showing the role of sleep spindles in neurodevelopmental disorders, few studies in humans directly compare clinical populations and even fewer studies track the developmental course of sleep spindles in typically and atypically developing children. Therefore, while, taken as a whole, alterations in sleep spindles and their features are associated with neurodevelopmental disorders, it is still unclear whether (1) there are disorder-specific phenotypes that may inform disease course and treatment response and (2) the developmental course of such deficits (e.g., at what age do they emerge). As for the first point, as previously mentioned, reduced sleep spindle density is seen in (some) intellectual disabilities, ASD, and childhood onset schizophrenia; however, whether a direct comparison across these disorders will reveal more nuanced differences in sleep spindle is unknown. Furthermore, despite a general decline in sleep spindle activity in neurodevelopmental and neuropsychiatric disorders, a subsample of individuals do not show this decline. This likely reflects heterogeneity in disorders which is further supported by diversity of behavioral manifestations in patients with shared diagnosis. There are five diagnostic criteria for schizophrenia, but only two of them must be met to receive the diagnosis [147]. Therefore, two individuals with the same diagnosis may not share the same set of symptoms. Exemplary of this is in childhood onset schizophrenia (COS) in which there is a strong association between hallucinations and sleep spindle power [148, 149]. In rodents, illustration of this comes from the difference in spindle density and expression in different mouse models, e.g., GCLM KO, and the Gria KO -/- mice (see section above) [15].

As a result, these examples illustrate that treatments targeting the thalamocortical system may benefit a subset of patients and that sleep spindles may provide a window to study specific brain circuit alterations. Given the cardinal role of this circuit in neurodevelopmental disorders, as outlined above, we postulate that sleep spindles will be increasingly utilized as a biomarker in drug development and treatment efficacy in the next decade.

Moreover, the emphasis thus far has been on characterizing spindle parameters at a few electrodes. Additional sleep spindle activity metrics such as coherence (a measure of synchrony of sleep spindles across brain regions) or the temporal coupling between slow waves and sleep spindles could provide valuable information for differentiating across disorders and guiding treatment decisions. Given the homeostatic changes in sleep spindle frequency and incidence across the night, the dynamics of sleep spindle activity over the course of the night may provide additional insights into the functioning of the thalamocortical system.

Overall, rodent models of neurodevelopmental disorders have shed light on the neural basis of these disorders, but modeling mental disorders in animals, especially rodents, is difficult. Understanding the circuitry that controls sleep spindle characteristics could aid in understanding the neurodevelopmental deficits in many mental disorders. The thalamocortical system is conserved across species [150]; hence, animal models can be used to test new pharmacological therapies using cutting-edge predictive algorithms based on circuit activity. Using human EEG signals to identify biomarkers in patient cohorts is a back translation-human inspired approach and allows for the study of human EEG sleep spindle oscillation-related neural activity.

Because over 85% of sleep spindles are heritable, the sleep EEG may be a unique biomarker for neurodevelopmental disorders [58]. This is important during early adolescence when sleep neurophysiology and the brain change significantly. Genetics underlie most neurodevelopmental and neuropsychiatric disorders. Sleep spindles may link genetics to disorder onset. Many prominent models of psychopathology, such as the diathesis stress model and the differential susceptibility hypothesis of mental health, suggest that a genetic predisposition to a disorder may interact with environmental factors like maternal immune activation to cause a disorder such as schizophrenia. Alterations in sleep spindles may indicate genetic susceptibility to neurodevelopmental or psychiatric disorders, which may be exacerbated by environmental factors. However, much work is needed in this area. In longitudinal studies of high-risk children for neurodevelopmental disorders (e.g., infants with an ASD sibling), sleep spindles should be explicitly tested for predictive value. Large-scale studies are needed to determine which spindle features (e.g., density) change and how.

To summarize, research suggests that disruptions to the thalamocortical circuit may contribute to the development and maintenance of neuropsychiatric disorders. Additionally, sleep spindles are a promising tool for assessing thalamocortical integrity, with important implications for the diagnosis and treatment of these complex neuro-developmental disorders. More research is needed to better understand the specific underlying mechanisms, both common and unique to these disorders, to develop better-targeted treatments for these conditions.

In the first column the first author, journal and year of publication are cited in addition to the sample size and age (range and mean when available). The columns amplitude, frequency, duration, and density refer to algorithm of visual scoring that have characterized individual sleep spindles. We note here that there is some variation among publications how these indices are quantified. Sigma power refers to spectral power in the sigma band during NREM sleep, which is associated with sleep spindles. Again, the definition of this frequency band is publication dependent. *NA* this metric was not used in the cited publication, *no change* there was no statistically significant difference between the control and experimental group, *reduced* the metric was reduced in the experimental in comparison to the control group.

## Declarations

The Section Editor for the topical collection Sleep and Development is Leila Tarokh. Please note that Section Editor, Dr. Tarokh was not involved in the editorial process of this article as she is a co-author.
